# Estimating the burden of mycetoma in Sudan for the period 1991–2018 using a model-based geostatistical approach

**DOI:** 10.1371/journal.pntd.0010795

**Published:** 2022-10-14

**Authors:** Rowa Hassan, Jorge Cano, Claudio Fronterre, Sahar Bakhiet, Ahmed Fahal, Kebede Deribe, Melanie Newport

**Affiliations:** 1 Mycetoma Research Centre, Soba University Hospital, University of Khartoum, Khartoum, Sudan; 2 Department of Global Health and Infection, Brighton and Sussex Medical School, Brighton, United Kingdom; 3 Expanded Special Project for Elimination of Neglected Tropical Diseases, WHO Regional Office for Africa, Brazzaville, Democratic Republic of the Congo; 4 Centre for health informatics, computing, and statistics, Lancaster Medical School, Lancaster University, Lancaster, United Kingdom; 5 Children’s Investment Fund Foundation, Addis Ababa, Ethiopia; 6 School of Public Health, College of Health Sciences, Addis Ababa University, Addis Ababa, Ethiopia; Albert Einstein College of Medicine, UNITED STATES

## Abstract

Mycetoma is widespread in tropical and subtropical regions favouring arid areas with low humidity and a short rainy season. Sudan is one of the highly endemic countries for mycetoma. Estimating the population at risk and the number of cases is critical for delivering targeted and equitable prevention and treatment services. In this study, we have combined a large dataset of mycetoma cases recorded by the Mycetoma Research Centre (MRC) in Sudan over 28 years (1991–2018) with a collection of environmental and water and hygiene-related datasets in a geostatistical framework to produce estimates of the disease burden across the country. We developed geostatistical models to predict the number of cases of actinomycetoma and eumycetoma in areas considered environmentally suitable for the two mycetoma forms. Then used the raster dataset (gridded map) with the population estimates for 2020 to compute the potentially affected population since 1991. The geostatistical models confirmed this heterogeneous and distinct distribution of the estimated cases of eumycetoma and actinomycetoma across Sudan. For eumycetoma, these higher-risk areas were smaller and scattered across Al Jazirah, Khartoum, White Nile and Sennar states, while for actinomycetoma a higher risk for infection is shown across the rural districts of North and West Kurdufan. Nationally, we estimated 63,825 people (95%CI: 13,693 to 197,369) to have been suffering from mycetoma since 1991 in Sudan,51,541 people (95%CI: 9,893–166,073) with eumycetoma and 12,284 people (95%CI: 3,800–31,296) with actinomycetoma. In conclusion, the risk of mycetoma in Sudan is particularly high in certain restricted areas, but cases are ubiquitous across all states. Both prevention and treatment services are required to address the burden. Such work provides a guide for future control and prevention programs for mycetoma, highly endemic areas are clearly targeted, and resources are directed to areas with high demand.

## Author summary

Mycetoma is a chronic infection that is recently been labelled as a neglected tropical disease (NTD) by the World Health Organization (WHO). The epidemiological features of mycetoma are not well described. The prevalence of the disease are globally underestimated. In Sudan, there are few studies reporting prevalence. In order to estimate mycetoma burden, we combined a large dataset of mycetoma cases recorded by the Mycetoma Research Centre (MRC) in Sudan with a collection of environmental and water and hygiene-related datasets in a geostatistical framework to produce estimates of the disease burden across the country. Our geostatistical models fitted based on the eumycetoma and actinomycetoma cases recorded by the MRC in Sudan between 1991 and 2018, have shown a spatially heterogeneous and distinct distribution of both mycetoma forms across this endemic country. According to our predictions, most of the eumycetoma cases would have occurred around the Khartoum area and Al Jazirah state and most of the actinomycetoma cases would have concentrated in the rural North and West Kurdufan states. Our findings here have several implications for clinical and public health practices. Both prevention and treatment services are required to address the burden. Such work provides a guide for future control and prevention programs for mycetoma, highly endemic areas are clearly targeted, and resources are directed to areas with high demand. Moreover, medical personnel will have high levels of suspicion for mycetoma in areas with high burden and cases will be adequately diagnosed and managed within their communities without the need to seek medical treatment in centralized medical facilities, in turn reducing the financial burden for mycetoma patients.

## Introduction

Mycetoma is a chronic debilitating infection designated a neglected tropical disease (NTD) by the World Health Organization (WHO) [1,2]. It mainly presents as a painless swelling with sinus tracts that discharge granules encapsulating the causative organisms [3]. The infection mainly affects the lower extremities, but any body parts can be involved [2,4,5]. Mycetoma presents in two forms according to the causative agent; eumycetoma caused by fungal agents and actinomycetoma caused by a group of filamentous bacteria [6–9]. Young adult males are mostly affected by this condition, although all age groups and gender are susceptible [10,11]. Affected communities are usually underprivileged with poor access to education and safe sanitation [12,13]. Farmers and shepherds are the most commonly exposed due to the nature of their occupations and interaction with the environment [14,15].

Mycetoma is widespread in tropical and subtropical regions favouring arid areas with low humidity and a short rainy season [16–18]. It has been reported from Africa, South and Central America, and Asia [19]. Sudan is one of the highly endemic countries for mycetoma with a massive impact on patients, the community, and the health system. Most mycetoma cases in Sudan are caused by the fungal form (eumycetoma), and the primary causative agent is *Madurella mycetomatis* [20,21]. The medical literature highlights the role of thorn pricks in the pathogenesis of mycetoma by stressing the association of mycetoma occurrence with trauma, especially from acacia trees thorns. Frequently, thorns are identified impeded in mycetoma lesions during surgery, supporting this theory. However, it is still unclear whether the trauma creates a portal of entry for pathogenic organisms resident in soil or the acacia thorns are infected, causing direct inoculation [21,22].

The epidemiological features of mycetoma are not well described. The incidence and prevalence of the disease are globally underestimated, and most of the reported cases are based on hospital records and short prevalence surveys conducted locally [23,24]. Since the middle of the last century, much effort was made to estimate the mycetoma burden in Sudan accurately. Abbott’s study in 1952 estimated the prevalence of mycetoma to be 4.6 per 100,000 inhabitants based on a cohort of 1,231 mycetoma patients admitted to hospitals throughout the country [25]. More recently, in 2014, a large meta-analysis conducted by van de Sande *et al*. estimated that the mycetoma prevalence in Sudan was 1.81 cases per 100,000 inhabitants, although the authors acknowledged this could be much higher in some villages [26].

Estimating the population at risk and number of cases cases is critical for delivering targeted and equitable prevention and treatment services, planning control and elimination programs, and implementing tailored case-finding and surveillance. Estimates of number of cases and population at risk are typically obtained through routine disease surveillance (secondary data sources), house-to-house case searches or large-scale surveys. For diseases of low prevalence such as mycetoma, routinely collected data is not reliable enough to produce estimates of the disease burden since it underestimates their actual impact and distribution. On the other hand, although house-to-house and large-scale surveys can deliver more accurate estimates, they tend to be unfeasible because of their high cost and logistic needs. Nowadays, geospatial modelling techniques for predicting the distribution and burden of diseases have proven critical to produce reliable estimates, especially in low-income countries with limited resources [27–30]. In this study, we have combined a large dataset of mycetoma cases recorded by the Mycetoma Research Centre (MRC) in Sudan over 28 years (1991–2018) with a collection of environmental and sanitation-related datasets in a geostatistical framework to produce estimates of the disease burden across the country. Number of cases estimates were generated separately for the bacterial (actinomycetoma) and fungal (eumycetoma) forms of mycetoma.

## Materials and methods

### Records of mycetoma cases

The data included in this study were extracted from the patient database compiled by the Mycetoma Research Centre (MRC) at Soba University Hospital in Khartoum (Sudan) from 1991–2018. This centre was established in 1991 to manage mycetoma patients attending from all states of Sudan, and more than 10,000 cases have been registered at the centre since it opened [25,31].

**Fig 1:**
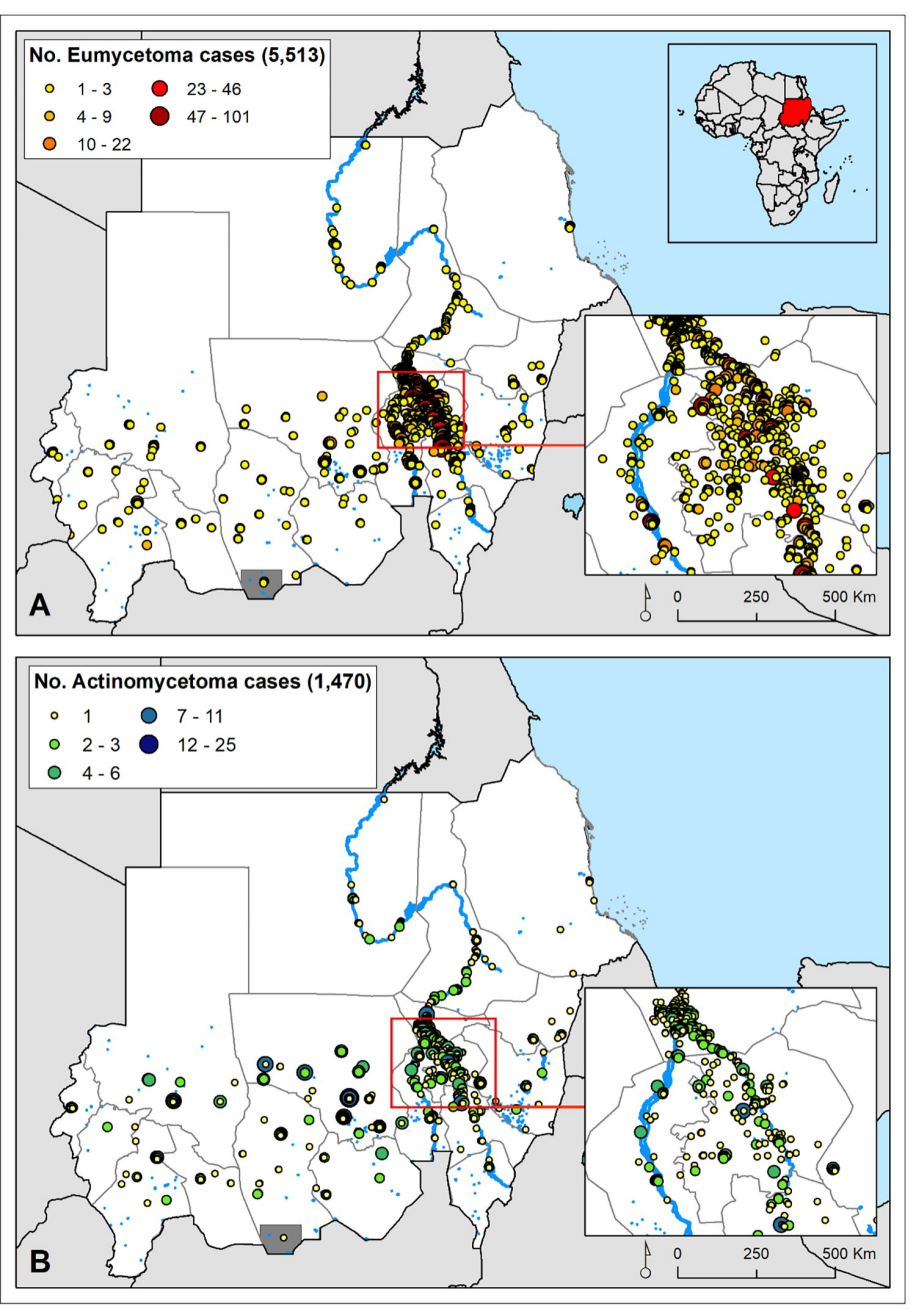
Distribution of eumycetoma (A) and actinomycetoma (B) cases recorded by the Mycetoma Research Centre from 1991 to 2018.

The variables extracted from the database included patients’ demographic characteristics, details of their clinical presentation and diagnosis, and their location of origin using patients’ addresses, which were then used to find the most accurate location, namely geographical coordinates, for all the recorded mycetoma cases (Fig 1).

### Explanatory variables

Geostatistical models based on the counts of actinomycetoma and eumycetoma cases were constructed separately using two independent variables potentially associated with the occurrence and distribution of mycetoma: environmental suitability and an indicator of poor hygiene conditions [32]. We used gridded surfaces of predicted environmental suitability for both types of mycetoma as modelled previously (S1 Fig) [17]. Environmental suitability for mycetoma across Sudan was modelled using a combination of linear regression and machine learning algorithms. These algorithms were used to identify the environmental risk factors associated with the occurrence of mycetoma cases, and subsequently established the likelihood for the occurrence of mycetoma based on the values recorded for each environmental factor at every area of 1sq-km. We also obtained a continuous estimate of household access to unimproved sanitation services by WHO/UNICEF Join Monitoring Program by 2017. As defined by this program, an "improved" sanitation facility is one that “safely separates excreta and wastewater from human contact either by safe containment and disposal in situ or by safe transport and treatment off-site. Unimproved latrine, open defecation, well without a pump and surface water are considered unimproved sources of sanitation. [33]. (S2 Fig) This gridded map displaying the estimated percentage of households using unimproved sanitation is based on a Bayesian geostatistical model developed using a collection of environmental and socio-economic data and data from 600 sources across more than 88 low-income and middle-income countries (LMICs) [32].

### Geostatistical modelling to estimate mycetoma burden

The records of mycetoma cases and modelled environmental suitability and accessibility to unimproved sanitation were combined within a geostatistical framework. The records of geolocated mycetoma cases were aggregated on a spatial grid of 1 sq-km resolution across the country. Thus, we modelled counts of mycetoma cases per 1 km^2^ for 1991–2018 and took the estimated gridded population at the same spatial resolution for 2020 into account. Due to the lack of reliable census data for all the populated areas across Sudan, we obtained gridded continuous estimates of the total population for 2020 from the WorldPop project [34,35]. Because of the patchy distribution of the population in Sudan, we opted for using the constrained version of modelled estimates at 100 metres resolution [36,37]. This method only generates estimates within areas containing built settlements (S3 Fig). We aggregated the population estimates to a grid of 1 sq-km resolution to match the geographical aggregation for mycetoma cases and the covariates.

We developed geostatistical models to predict the number of actinomycetoma and eumycetoma cases in areas considered environmentally suitable for the two mycetoma forms, as delineated by a previous modelling exercise across Sudan (S4 and S5 Figs) [17]. We let mycetoma risk depend on the predicted environmental suitability and household level utilization of unimproved sanitation values obtained in the previous step. We included spatial random effects to account for spatial variation in mycetoma cases between locations of origin that was not explained by the explanatory variables, and independent random effects to account for potential overdispersion. We validated the models using a variogram-based procedure that tests the compatibility of the adopted spatial structure with the data. More details are provided in S1 Text. The analysis was carried out using the R package *PrevMap v 1*.*5*.*3* [38], which implements geostatistical models’ parameter estimation and spatial prediction using Monte Carlo Maximum Likelihood [39]. This model was applied to produce predictions of the number of eumycetoma and actinomycetoma cases since 1991 at 1km^2^ spatial resolution and probability maps of exceeding a 50 per 10,000 people and 5 per 10,000 people cases thresholds for eumycetoma and actinomycetoma, respectively. The reason two different threshold were used eumycetoma is more common in Sudan compared to actinomycetoma. We checked the validity of the assumed covariance model for the spatial correlation using the Monte Carlo algorithm and empirical semi-variogram as described in S7 Fig. Additionally, maps of 95% confidence intervals for the number of cases were generated for each 1 sq-km grid location.

We used the raster dataset (gridded map) with the population estimates for 2020 downloaded from the WorldPop project to compute the potentially affected population since 1991. An output raster dataset computing the estimated number of eumycetoma and actinomycetoma cases per grid cell was obtained by multiplying the 1km^2^ raster dataset of predictive number of cases with the corresponding grided map with the total population estimated per 1 sq-km pixel for built-up areas. The same procedure was used to estimate the uncertainty range of the affected population using the gridded maps of 95% confidence interval (CI) for predicted number of cases. These raster datasets were then used to extract the aggregate number of people with mycetoma and uncertainty range by administrative area (district and states).

## Results

### Mycetoma records: General description

The modelled data included 7,812 unique points obtained from patients seen at the MRC in the period 1991–2018, and they came from all eighteen states of Sudan. The study included 5,513 patients (79%) with confirmed eumycetoma and 1,470 patients (21%) with actinomycetoma. Most of the mycetoma patients were from Al Jazirah State (34.4%) and Khartoum State (14.5%) (Fig 1). Further details of the patients’ geographical distribution have been published elsewhere [17].

### Predicted number of mycetoma cases and burden estimation

**Fig 2:**
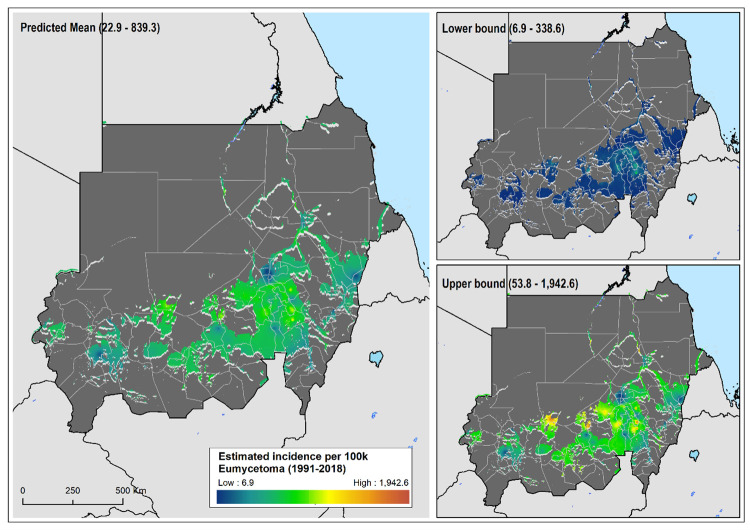
Estimated eumycetoma cases per 100,000 inhabitants between 1991 and 2018; mean predicted number of cases and, lower and upper 95% CI bounds. Areas considered environmentally unsuitable for the occurrence of eumycetoma as predicted by environmental model have been excluded.

**Fig 3:**
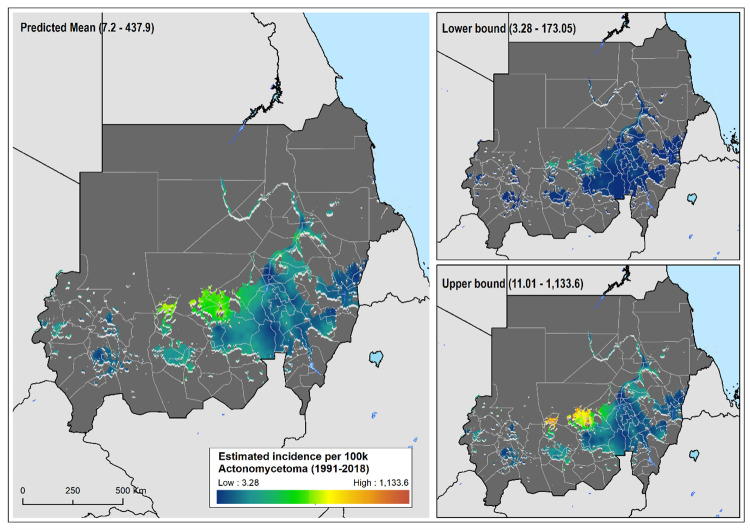
Estimated actinomycetoma cases per 100,000 inhabitants between 1991 and 2018; mean predicted number of cases and, lower and upper 95% CI bounds. Areas considered environmentally unsuitable for the occurrence of actinomycetoma as predicted by environmental model have been excluded.

**Fig 4:**
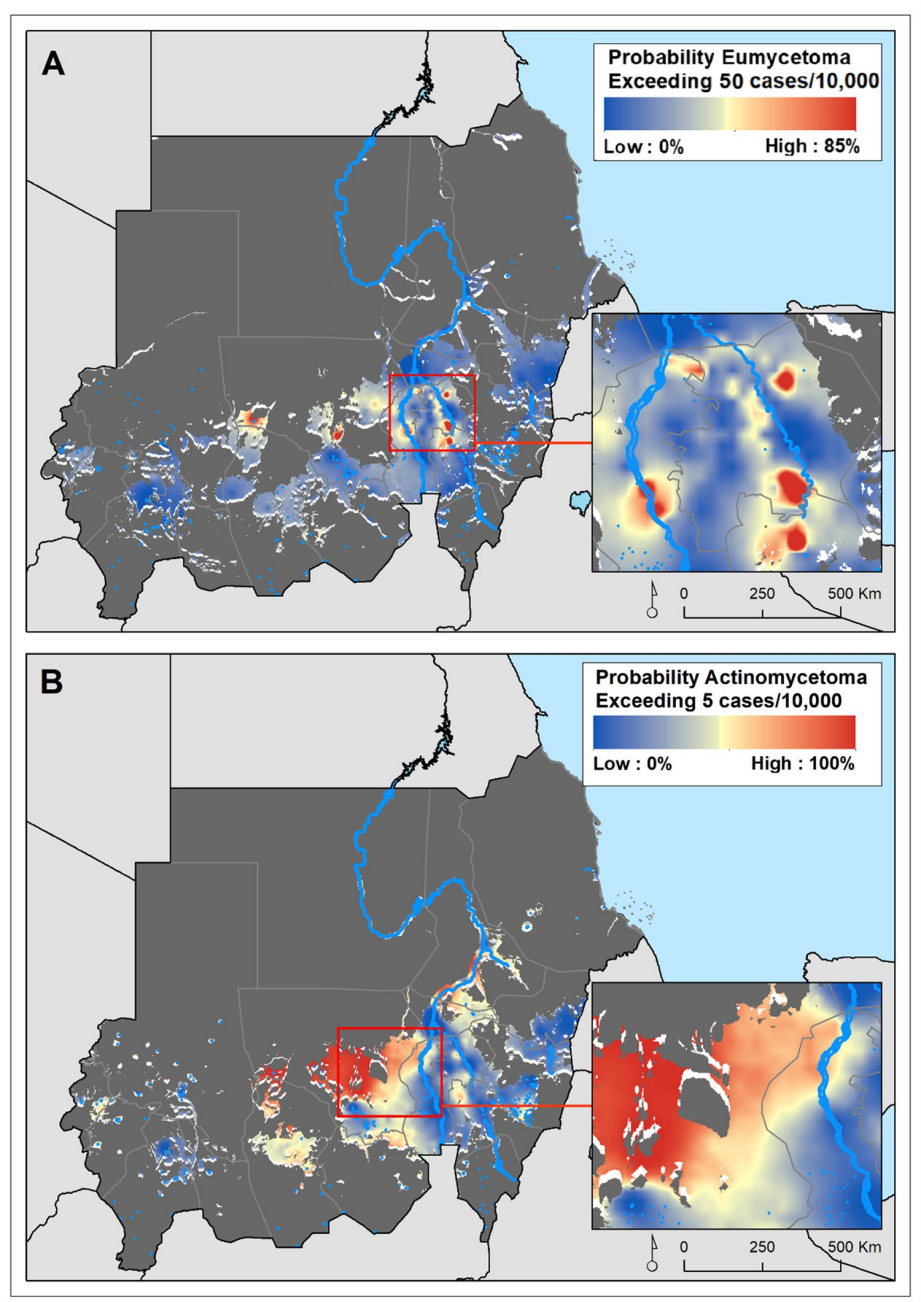
Maps displaying the probability of exceeding 50 cases and 5 cases per 1,000 inhabitants, for eumycetoma and actinomycetoma respectively, since 1991 in Sudan.

The relative risk of eumycetoma was over 4-fold higher in the localities around the Khartoum area, most of them in the state of Al Jazirah, than was expected across Sudan for the study period, whereas those at higher risk of actinomycetoma were from the North Kurdufan state (S6 Fig). The geostatistical models confirmed this heterogeneous and distinct distribution of the estimated number of eumycetoma and actinomycetoma cases across Sudan (Figs 2 & 3). For eumycetoma, these higher-risk areas were smaller and scattered across Al Jazirah, Khartoum, White Nile and Sennar states, while for actinomycetoma a higher risk for infection is shown across the rural districts of North and West Kurdufan. The data also clearly showed the exceedance probability of number of cases rate of 50 per 10,000 inhabitants and 5 per 10,000 inhabitants for eumycetoma and actinomycetoma, respectively (Fig 4).

**Fig 5:**
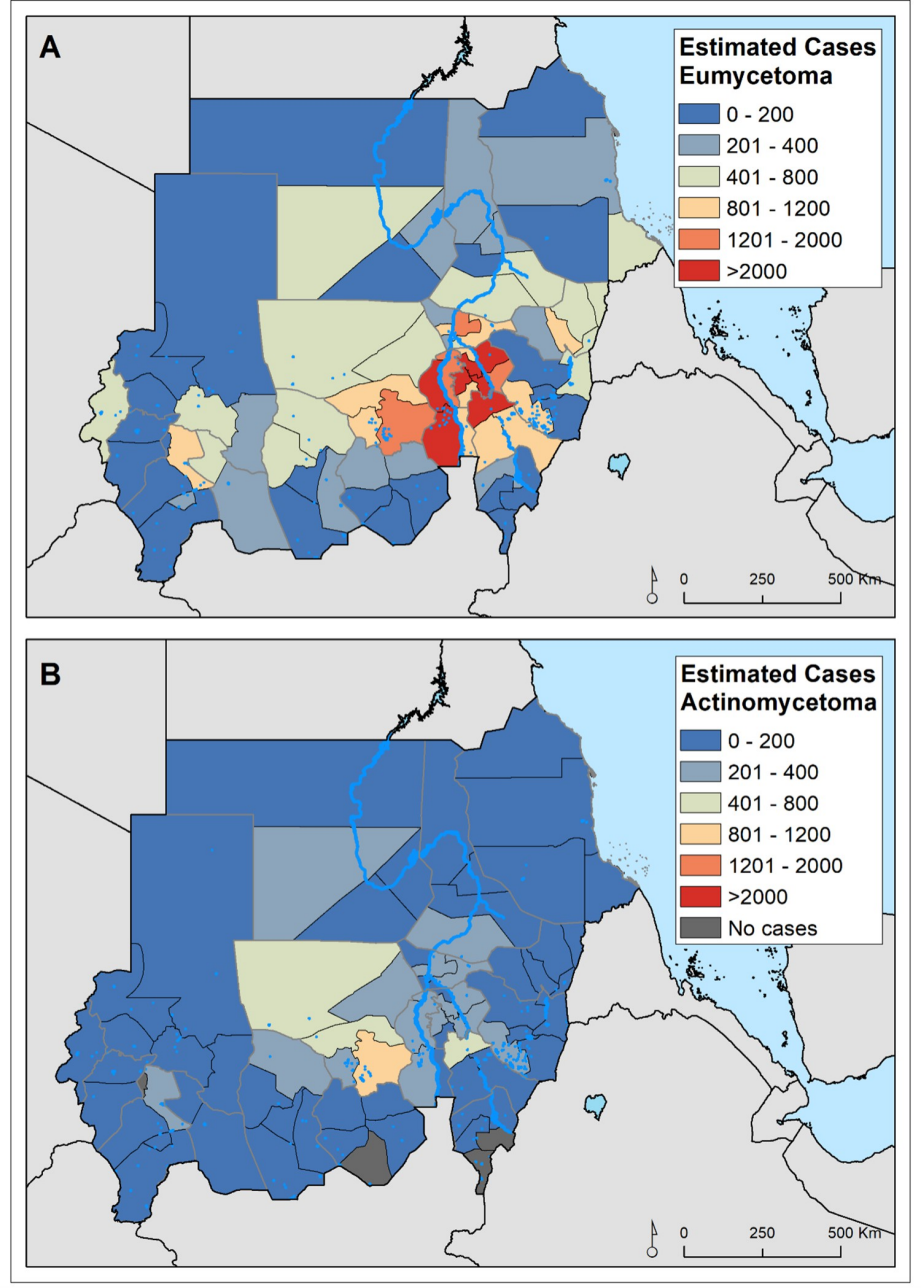
Estimated number of people that have eumycetoma (A) and actinomycetoma (B) between 1991 and 2018 as predicted by the fitted model-based geostatistical models.

Nationally, we estimated 63,825 people (95%CI: 13,693 to 197,369) to have been suffering from mycetoma since 1991 in Sudan (Tables 1 & 2): 51,541 people (95%CI: 9,893–166,073) with eumycetoma and 12,284 people (95%CI: 3,800–31,296) with actinomycetoma. Five regions (Al Jazirah, White Nile, North Kurdufan, Khartoum and Sennar) would have contributed over 66% of the total number of cases expected in the period 1991–2018. The greatest proportion (22%) of people affected by any form of mycetoma resided in the Al Jazirah state, although when differentiating by type of mycetoma, most of actinomycetoma cases would have occurred in the North Kurdufan state. The remaining thirteen states were predicted to have had less than 1,000 cases of mycetoma during the study period (Fig 5). S1 and S2 Tables provide district level estimates of the number of expected eumycetoma and actinomycetoma cases respectively.

**Table 1:** Estimation of eumycetoma cases by state in Sudan between 1991 and 2018.

State	Area predicted suitable (sq-km)	Estimated Eumycetoma Cases			
		No.	%	95% CI	
				Lower Bound	Upper Bound
Al Jazirah	22,307	12,263	23.8%	2,979	34,452
Al Qadarif	21,786	1,910	3.7%	297	6,689
Blue Nile	4,030	543	1.1%	88	1,854
Central Darfur	3,894	255	0.5%	31	970
East Darfur	12,964	1,046	2.0%	129	3,984
Kassala	28,963	3,205	6.2%	430	11,799
Khartoum	18,113	5,139	10.0%	1,231	14,837
North Darfur	18,683	1,163	2.3%	158	4,266
North Kurdufan	52,628	4,680	9.1%	696	16,582
Northern	6,696	989	1.9%	159	3,396
Red Sea	8,101	792	1.5%	102	2,999
River Nile	27,403	2,731	5.3%	464	9,176
Sennar	28,373	5,046	9.8%	1,110	15,271
South Darfur	13,169	1,547	3.0%	224	5,599
South Kurdufan	16,397	713	1.4%	96	2,62
West Darfur	5,983	506	1.0%	73	1,834
West Kurdufan	34,959	1,850	3.6%	238	6,910
White Nile	40,855	7,163	13.9%	1,388	22,834
**Total**	**365,304**	**51,541**		**9,893**	**166,073**

**Table 2:** Estimation of actinomycetoma cases by state in Sudan between 1991 and 2018.

State	Area predicted suitable (sq-km)	Estimated Actinomycetoma Cases			
		No.	%	95% CI	
				Lower Bound	Upper Bound
Al Jazirah	22,995	1,801	14.7%	719	3,794
Al Qadarif	33,525	489	4.0%	129	1,341
Blue Nile	2,341	118	1.0%	33	318
Central Darfur	2,850	89	0.7%	16	289
East Darfur	5,661	184	1.5%	45	543
Kassala	16,028	367	3.0%	103	978
Khartoum	15,910	1,351	11.0%	580	2,882
North Darfur	9,765	449	3.7%	113	1,291
North Kurdufan	69,740	2,826	23.0%	747	7,714
Northern	4,934	527	4.3%	139	1,392
Red Sea	2,549	123	1.0%	32	352
River Nile	23,565	869	7.1%	260	2,220
Sennar	25,218	729	5.9%	247	1,722
South Darfur	8,896	357	2.9%	91	1,014
South Kurdufan	9,498	161	1.3%	33	496
West Darfur	4,368	143	1.2%	40	39
West Kurdufan	22,912	545	4.4%	117	1,650
White Nile	42,463	1,156	9.4%	356	2,907
**Total**	**323,218**	**12,284**		**3,800**	**31,296**

In terms of validating the geostatistical models fitted, the variogram-based procedure conducted using the data simulated from the models led us to conclude that the observed data are compatible with the assumptions of an exponential correlation function and that the underlying spatial structure has been accounted for by the spatial fixed and random effects (S7 and S8 Figs).

## Discussion

To our knowledge, this is the first analysis to use geostatistical modelling to estimate the burden of mycetoma. The novelty of the current analysis is that we combined mycetoma case data with a collection of environmental factors to estimate the burden of mycetoma across Sudan. We have also provided the associated uncertainty interval to identify areas where further data collection is required to improve the estimates. Given the difference in aetiology and potential risk factors for each type of mycetoma, we have fitted separate models for eumycetoma and actinomycetoma. Our analysis can serve as a framework to estimate the global burden of mycetoma.

Our geostatistical models fitted based on the eumycetoma and actinomycetoma cases recorded by the MRC in Sudan between 1991 and 2018, have shown a spatially heterogeneous and distinct distribution of both mycetoma forms across this endemic country. According to our predictions, most of the eumycetoma cases would have occurred around the Khartoum area and Al Jazirah state, and most of the actinomycetoma cases would have concentrated in the rural North and West Kurdufan states. It has also showed that the number of mycetoma cases in Sudan, so far mostly estimated through hospital records and local prevalence surveys [25,26], is likely to be sizeably underestimated.

Al Jazirah and North Kurdufan states showed high estimates of mycetoma cases, which can be attributed to the nature of the economic activities of the population residing those areas. Most of the people there are sustained by arable farming or animal grazing, which increase the risk of contact with mycetoma causative organisms. Al Jazirah state had the biggest agriculture scheme in Sudan and North Kurdufan state is one of the biggest states in the production of gum Arabic.

Overall, the estimated number of eumycetoma cases is four times higher than actinomycetoma in Sudan and across the states. This is in agreement with what has been reported through clinical observations and reports where the number of eumycetoma dominates [25]. Nonetheless, there is no difference in proportions of areas suitable for the occurrence of both mycetoma forms. One six (17%) and 19% of the landmass of Sudan is suitable for the occurrence of actinomycetoma and eumycetoma respectively. For both types of mycetoma high environmental suitability was predicted in North Kurdufan and White Nile states.

The localities that had higher estimates of eumycetoma cases include, North Jazirah, Khartoum Bahri, Um Rawaba, Sennar and Ad Douiem. These localities have large populations, that manly depend on farming as an occupation. For actinomycetoma North Kurdufan state accounted for most of the cases and again Um Rawaba had the highest estimates.

Our modelling approach is not without limitations. First, we must assume there might be underlying geographical and temporal biases in the recorded number of mycetoma cases in the country, as all the records were collected by the Mycetoma Research Centre, which is located in Khartoum, since its inception in 1991. From the exploratory analysis of this dataset, presented elsewhere [4], the number of mycetoma cases confirmed by the MRC has steadily increased since 1991 and the vast majority are coming from rural and peri-urban areas near the Khartoum area (Fig 1). In order to account for this potential geographical bias, we adjusted the number of cases by the estimated population density in 2020, although there still remain some uncertainty on where the infection may have occurred due to most cases being diagnosed in advance stages of the disease. Neither was it possible to account for any existing temporal variation due to the uncertainty on when the infection took place, severe cases are more likely to be diagnosed in health facilities. Second, we did not account for any individual or household related risk factors such as work and social activities, that may also have driven the distribution and intensity of transmission [24]. We however included an estimate of poor sanitation coverage, assuming a higher risk in more deprived areas with limited access to protected sanitation. Lastly patients address might be not appropriate indicator for point of infection in contagious diseases as well as mobile communities. Nonetheless, in our case the population under study are less mobile, the incubation period for mycetoma infection is not clearly defined, hence, the location of origin or address could be an indicator of the source of the infection.

Our findings here have several implications for clinical and public health practices. Our analysis indicated that only slightly less than a quarter of the landmass of Sudan is suitable for the occurrence of mycetoma. Nonetheless, the cases are ubiquitous across all states of Sudan which can be attributed to the population movement in Sudan. This implies that clinicians working in Sudan should have high index of suspicion for mycetoma for people presenting with swelling and sinuses discharging grains. Expanding diagnostic and treatment services to at least high burden states is required to address the existing cases. On the other hand, public health interventions focusing on prevention should focus on states and areas suitable for the occurrence of mycetoma. To prevent this disabling disease, geographically targeted public health education and social mobilization are required. Mycetoma can be prevented by encouraging safe farming practices like wearing shoes that decrease the chance of trauma, organizing interaction with animals and building ‘human-friendly’ animal cages that avoid the use of thorny tree branches. The burden presented here warrants a mycetoma control program in Sudan, which should coordinate the treatment and prevention of the disease.

In conclusion the risk of mycetoma in Sudan is particularly high in certain restricted areas, but cases are ubiquitous across all states. Both prevention and treatment services are required to address the burden. Such work provides a guide for future control and prevention programs for mycetoma, highly endemic areas are clearly targeted, and resources are directed to areas with high demand. Moreover, medical personnel will have high levels of suspicion for mycetoma in areas with high burden and case will be adequately diagnosed and managed within their communities without the need to seek medical treatment in centralized medical facilities, in turn reducing the financial burden for mycetoma patients. Specialized mycetoma management centres can be established in communities where mycetoma is endemic which provide early case detection and management of mycetoma patients.

## Disclaimer

The views expressed in this publication are those of the author(s) and not necessarily those of the NIHR or the Department of Health and Social Care” from the Financial Disclosure Statement.

## Supporting information

S1 Text Formulation and validation of geostatistical Poisson model. (PDF)

S1 Fig Maps of environmental suitability for eumycetoma (A) and actinomycetoma (B) The results of the Monte Carlo validation procedure for the actinomycetoma model.
(PDF)

S2 Fig Estimated percentage of households accessing unimproved sanitation by 2017The results of the Monte Carlo validation procedure for the eumycetoma model. (PDF)

S3 Fig Map displaying estimated total population for Sudan in 2020 based on constrained methods Bar plot showing the increasing trends of mycetoma cases in Sudan since 1991 to 2018. (PDF)

S4 Fig Predicted occurrence of actinomycetoma form of mycetoma and uncertainty range across Sudan. (PDF)

S5 Fig Predicted occurrence of eumycetoma form of mycetoma and uncertainty range across Sudan. (PDF)

S6 Fig Relative risk estimated at district level for eumycetoma and actinomycetoma based on cases recorded by the Mycetoma Research Centre (Khartoum) during the period 1991–2018 in Sudan. (PDF)

S7 Fig The results of the Monte Carlo validation procedure for the actinomycetoma model. (PDF)

S8 Fig The results of the Monte Carlo validation procedure for the eumycetoma model. (PDF)

S9 Fig Bar plot showing the increasing trends of mycetoma cases in Sudan since 1991 to 2018. (PDF)

S1 Table Estimation of eumycetoma cases by district in Sudan since 1991. (PDF)

S2 Table Estimation of actinomycetoma cases by district in Sudan since 1991. (PDF)
