# Supplementary material for: Estimating the burden of mycetoma in Sudan for the period 1991–2018 using a model-based geostatistical approach
Source: PLoS Negl Trop Dis. 2022 Oct 14;16(10):e0010795. doi: 10.1371/journal.pntd.0010795 (PMC9604875; doi:10.1371/journal.pntd.0010795)
Supplement: S1 Text — (PDF) [file pntd.0010795.s001.pdf]

S1_Text. Formulation and validation of geostatistical Poisson model with a log link function of mycetoma (actinomycetoma and eumycetoma) count of cases recorded from 1991 to 2018.

Let Y_i denote the number of confirmed mycetoma cases at location x_i from 1991 to 2018 over a total population m (exposure) at each location as determined by 2020 population estimates. We then assume that, conditionally on a zero-mean spatial Gaussian process $S(x)$, the Y_i observations are generated from a Poisson distribution with mean $m\lambda$, where m is an offset accounting for the exposure population as per demographics estimates for 2020. A canonical log link is used, thus the linear predictor assumes the form

$$\log\left\{\frac{\lambda(x_i)}{m(x_i)}\right\} = \beta_0 + \beta_1 EnvSuit(x_i) + \beta_2 UnimpSanitation(x_i) + S(x_i)$$

where the explanatory in the above equation is the modelled environmental suitability and estimates (percentage) of households using unimproved sanitation at location x_i.

We model the Gaussian process *S(x)* using an isotropic and stationary exponential covariance function given by

$$Cov\{S(x), S(x')\} = \sigma^2 exp\{-||x - x'||/\phi\}$$

Where $||x - x'||$is the Euclidean distance between x and x', σ^2 is the variance of *S(x)* and ϕ is a scale parameter that regulates how fast the spatial correlation decays to zero for increasing distance.

To check the validity of the adopted exponential correlation function for the spatial random effects *S(x)*, we carry out the following Monte Carlo algorithm.

1. Simulate a Poisson geostatistical dataset at observed locations x_i by plugging-in the maximum likelihood estimates from the fitted model.
2. Estimate the unstructured random effects Z_i from a non-spatial Poisson mixed model obtained by setting $S(x) = 0$ for all locations x.
3. Use the estimates for Z_i from the previous step to compute the empirical variogram.
4. Repeat steps 1 to 3 for 10,000 times.
5. Use the resulting 1,000 variograms to compute the 95% tolerance bandwidth under the hypothesis that the analysed data were generated by the fitted model. If the empirical variogram from the original data, obtained as in step 2, lies within 95% bandwidth, we then conclude that we do not find evidence against the assumption of an exponential correlation function for *S(x)*.
