# Supplementary material for: Estimating the burden of mycetoma in Sudan for the period 1991–2018 using a model-based geostatistical approach
Source: PLoS Negl Trop Dis. 2022 Oct 14;16(10):e0010795. doi: 10.1371/journal.pntd.0010795 (PMC9604875; doi:10.1371/journal.pntd.0010795)
Supplement: S2 Fig — (PDF) [file pntd.0010795.s003.pdf]

S2_Fig. Estimated percentage of households accessing unimproved sanitation by 2017, as published by Deshpande et al. 2020 [2]. This gridded map displaying the estimated percentage of households using unimproved sanitation is based on a Bayesian geostatistical model developed using a suite of environmental and socio-economic data and data from 600 sources across more than 88 low-income and middle-income countries (LMICs).

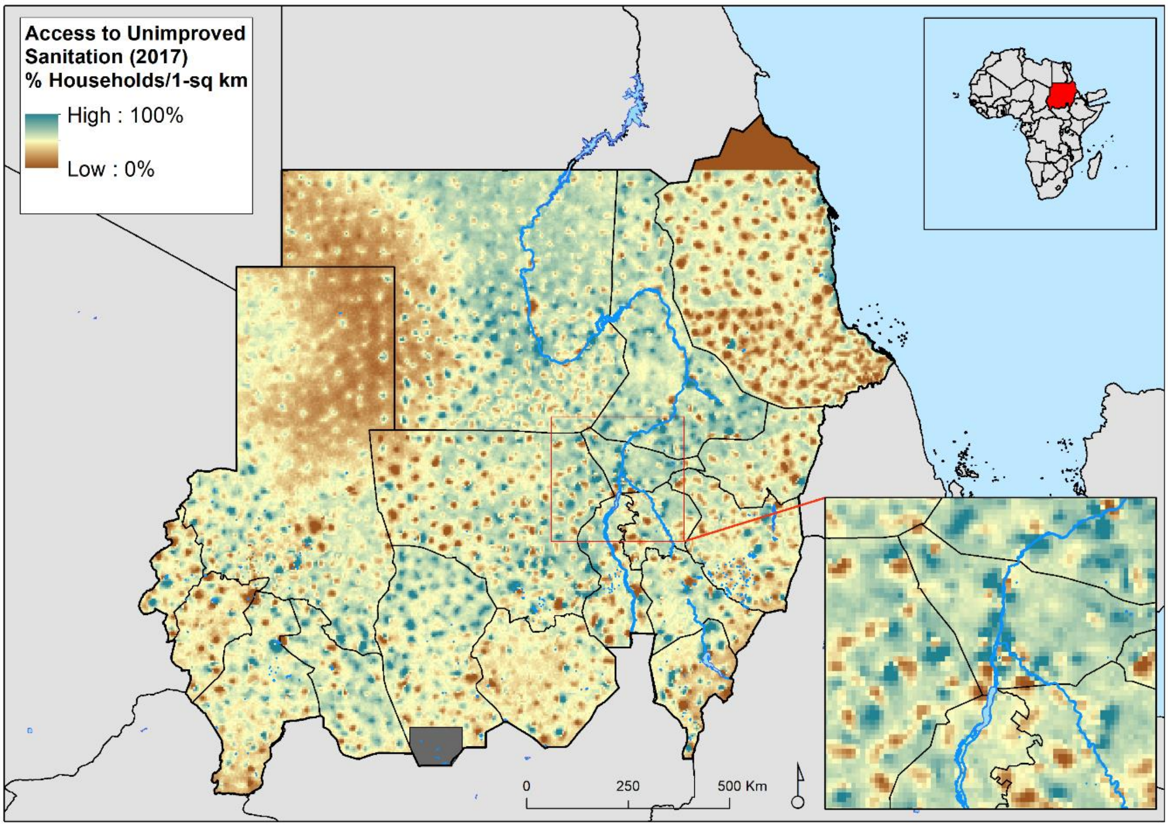

References

1. Hassan R, Simpson H, Cano J, Bakhiet S, Ganawa E, Argaw D, et al. Modelling the spatial distribution of mycetoma in Sudan. Trans R Soc Trop Med Hyg. 2021;115(10):1144-52. Epub 2021/05/27. doi: 10.1093/trstmh/trab076. PubMed PMID: 34037803; PubMed Central PMCID: PMCPMC8486737.

2. Deshpande A, Miller-Petrie MK, Lindstedt PA, Baumann MM, Johnson KB, Blacker BF, et al. Mapping geographical inequalities in access to drinking water and sanitation facilities in low-income and middle-income countries, 2000-17. The Lancet Global Health. 2020;8(9):e1162-e85. doi: 10.1016/S2214-109X(20)30278-3.

3. Reed FJ, Gaughan AE, Stevens FR, Yetman G, Sorichetta A, Tatem AJ. Gridded Population Maps Informed by Different Built Settlement Products. Data. 2018;3(3). doi: 10.3390/data3030033.
