# Supplementary material for: Estimating the burden of mycetoma in Sudan for the period 1991–2018 using a model-based geostatistical approach
Source: PLoS Negl Trop Dis. 2022 Oct 14;16(10):e0010795. doi: 10.1371/journal.pntd.0010795 (PMC9604875; doi:10.1371/journal.pntd.0010795)
Supplement: S4 Fig — (PDF) [file pntd.0010795.s005.pdf]

S4_Fig. Predicted occurrence of actinomycetoma form of mycetoma and uncertainty range across Sudan, as published by Rowa et al. 2021 [1]. This binary maps were generated from the predicted environmental suitability based on a cut-off above which the occurrence of actinomycetoma is highly likely. Optimal threshold was fitted to get better trade-off between sensitivity, specificity and proportion correctly classified (PCC).

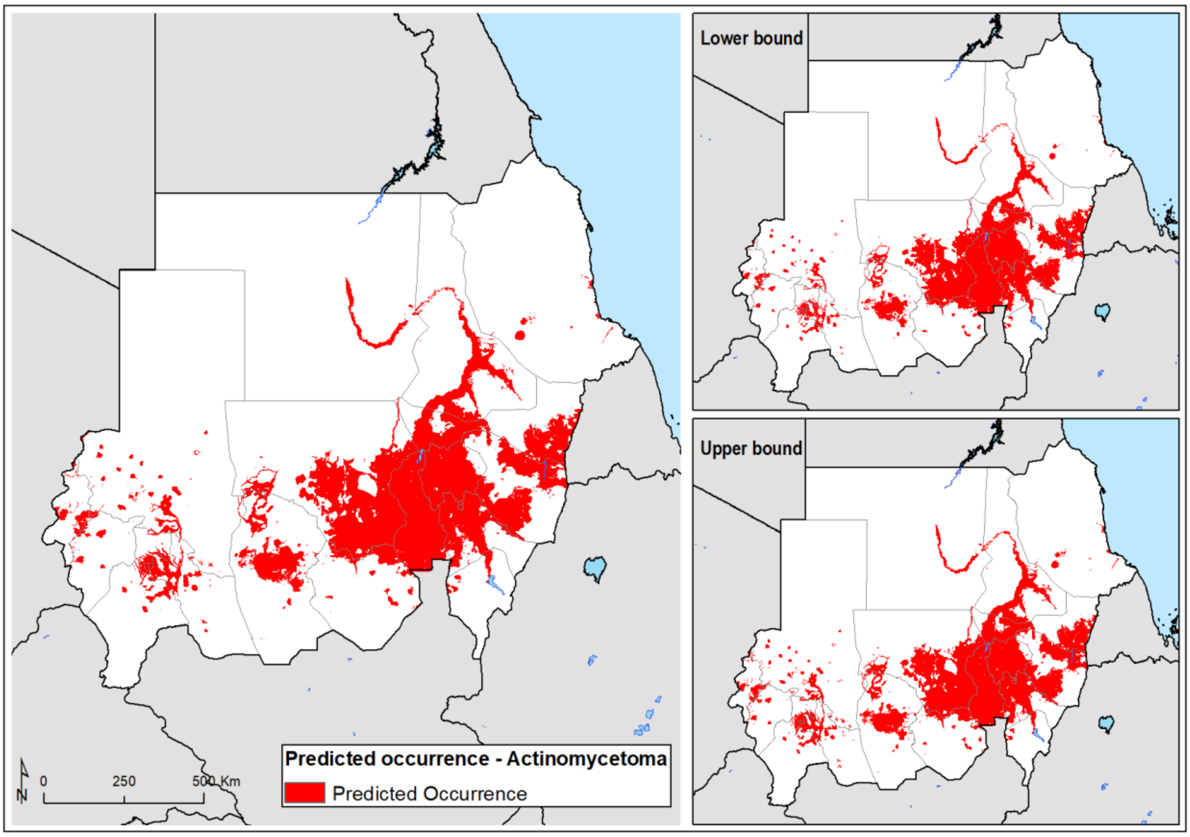

References

1. Hassan R, Simpson H, Cano J, Bakhiet S, Ganawa E, Argaw D, et al. Modelling the spatial distribution of mycetoma in Sudan. Trans R Soc Trop Med Hyg. 2021;115(10):1144-52. Epub 2021/05/27. doi: 10.1093/trstmh/trab076. PubMed PMID: 34037803; PubMed Central PMCID: PMCPMC8486737.

2. Deshpande A, Miller-Petrie MK, Lindstedt PA, Baumann MM, Johnson KB, Blacker BF, et al. Mapping geographical inequalities in access to drinking water and sanitation facilities in low-income and middle-income countries, 2000-17. The Lancet Global Health. 2020;8(9):e1162-e85. doi: 10.1016/S2214-109X(20)30278-3.

3. Reed FJ, Gaughan AE, Stevens FR, Yetman G, Sorichetta A, Tatem AJ. Gridded Population Maps Informed by Different Built Settlement Products. Data. 2018;3(3). doi: 10.3390/data3030033.
