# Supplementary material for: Estimating the burden of mycetoma in Sudan for the period 1991–2018 using a model-based geostatistical approach
Source: PLoS Negl Trop Dis. 2022 Oct 14;16(10):e0010795. doi: 10.1371/journal.pntd.0010795 (PMC9604875; doi:10.1371/journal.pntd.0010795)

S6_Fig. Relative risk estimated at district level for eumycetoma and actinomycetoma based on cases recorded by the Mycetoma Research Centre (Khartoum) during the period 1991 – 2018 in Sudan. The relative risk of mycetoma for each district was calculated as the ratio of the observed number of event over the expected number, taking the distribution of cases and population into account.

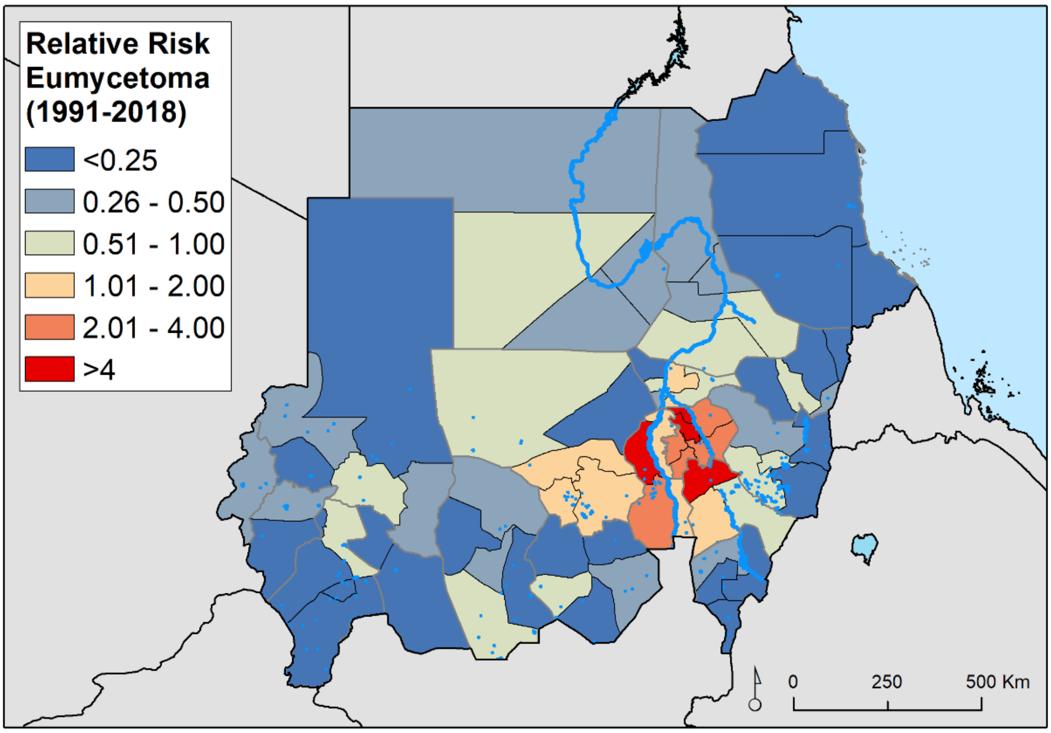

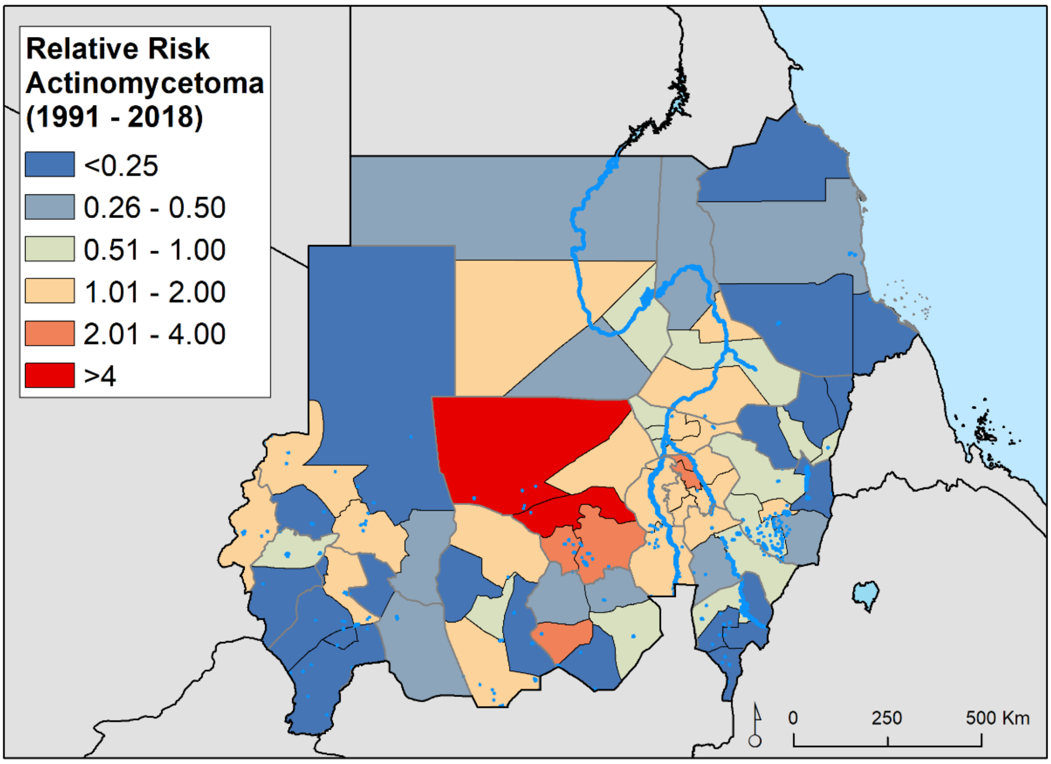

Supplement: S6 Fig — (PDF) [file pntd.0010795.s007.pdf]
