# Supplementary material for: Estimating the burden of mycetoma in Sudan for the period 1991–2018 using a model-based geostatistical approach
Source: PLoS Negl Trop Dis. 2022 Oct 14;16(10):e0010795. doi: 10.1371/journal.pntd.0010795 (PMC9604875; doi:10.1371/journal.pntd.0010795)

S8_Fig. The results of the Monte Carlo validation procedure for the eumycetoma model. The solid line is the observed variogram and the shaded area corresponds to the 95% bandwidth. The results lead us to conclude that the data are compatible with the assumption of an exponential spatial correlation function.

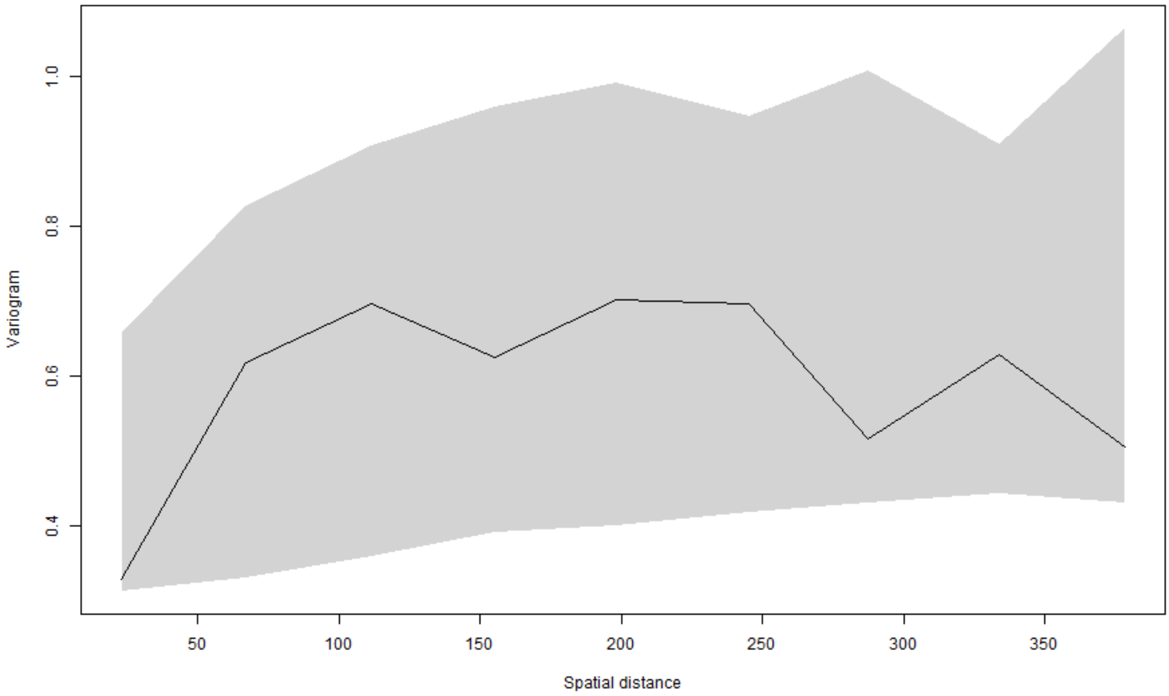

Supplement: S8 Fig — (PDF) [file pntd.0010795.s009.pdf]
