# Supplementary figures and images for: Estimating the burden of mycetoma in Sudan for the period 1991–2018 using a model-based geostatistical approach
Source: PLoS Negl Trop Dis. 2022 Oct 14;16(10):e0010795. doi: 10.1371/journal.pntd.0010795 (PMC9604875; doi:10.1371/journal.pntd.0010795)

**S9\_Fig.** Bar plot showing the increasing trends of mycetoma cases in Sudan since 1991 to 2018.

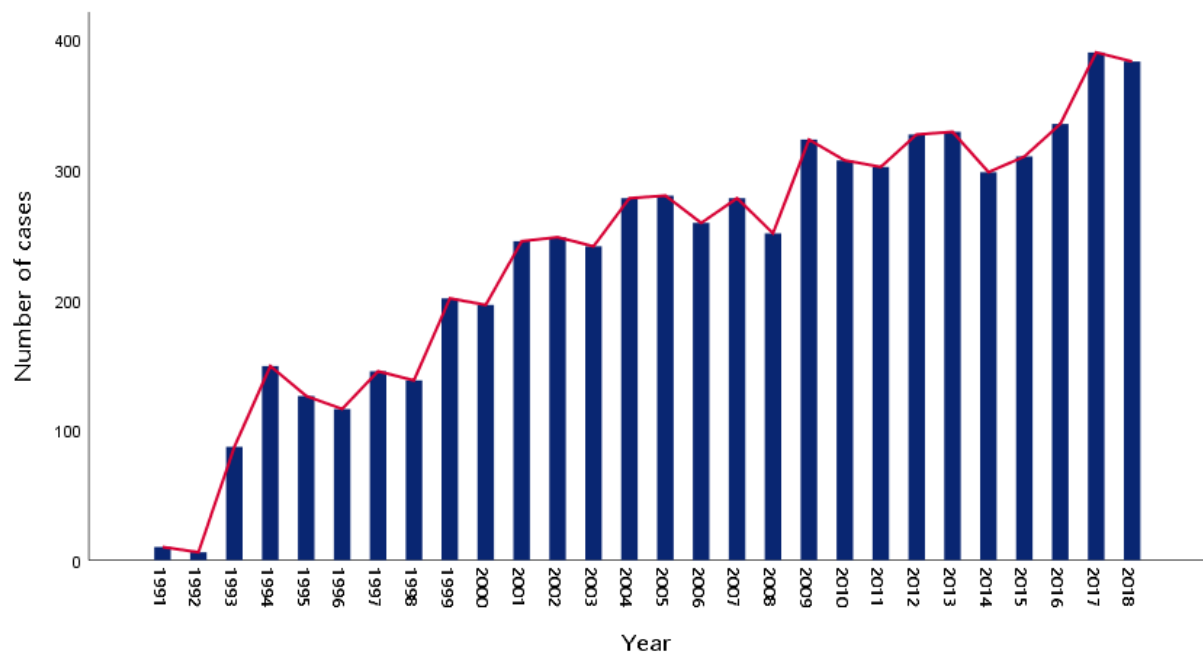

Supplement: S9 Fig — (PDF) [file pntd.0010795.s010.pdf]
