# Supplementary material for: Estimating the burden of mycetoma in Sudan for the period 1991–2018 using a model-based geostatistical approach
Source: PLoS Negl Trop Dis. 2022 Oct 14;16(10):e0010795. doi: 10.1371/journal.pntd.0010795 (PMC9604875; doi:10.1371/journal.pntd.0010795)
Supplement: S1 Table — (PDF) [file pntd.0010795.s011.pdf]

S1_Table. Estimation of eumycetoma cases by district in Sudan since 1991

State	District	Area predicted suitable (sq-km)	Estimated Eumycetoma Cases		
			No.	95% CI	
				Lower Bound	Upper Bound
Al Jazirah	Al Kamlin	1,618	1,144	305	3,063
Al Jazirah	Al Mahagil	3,610	2,008	470	5,754
Al Jazirah	East al Gazera	3,393	1,241	289	3,561
Al Jazirah	North al Gazera	2,950	2,400	630	6,444
Al Jazirah	Sharq al Gazera	5,072	2,177	515	6,222
Al Jazirah	South al Gazera	2,863	1,054	210	3,241
Al Jazirah	Um Al Gura	2,801	2,239	560	6,167
Al Qadarif	Al Faw	6,596	169	22	637
Al Qadarif	Al Fushqa	7,153	426	57	1,570
Al Qadarif	Al Gadaref	801	170	37	499
Al Qadarif	Al Galabat	2,065	180	22	684
Al Qadarif	Al Rahd	5,171	965	159	3,299
Blue Nile	Ad Damazin	2,703	318	53	1,079
Blue Nile	Al Kurumik	157	10	1	38
Blue Nile	Al Roseires	823	181	30	607
Blue Nile	Baw	346	34	4	130
Blue Nile	Geissan	1	-	-	-
Central Darfur	Mukjar	478	62	7	243
Central Darfur	Zallingi	3,416	193	24	727
East Darfur	Al Deain	4,192	265	38	976
East Darfur	Nyala	8,772	781	91	3,008
Kassala	Al Gash	10,386	746	90	2,834
Kassala	Hamashkorieb	3,161	775	84	3,067
Kassala	Kassala	3,149	504	84	1,690
Kassala	Nahr Atbara	7,891	937	140	3,323
Kassala	Seteet	4,376	243	32	885
Khartoum	Karary	1,578	301	48	1,040
Khartoum	Khartoum	346	608	196	1,459
Khartoum	Khartoum Bahri	4,936	1,324	313	3,841
Khartoum	Omdurman	1,574	842	224	2,298
Khartoum	Sharg En Nile	7,585	1,104	206	3,548
Khartoum	South Khartoum	981	640	169	1,718
Khartoum	Um Badda	1,113	320	75	933
North Darfur	Al Fasher	5,823	601	91	2,118
North Darfur	Kabkabiya	255	130	14	500
North Darfur	Kutum	1,632	22	4	73
North Darfur	Mellit	3,395	146	16	573

State	District	Area predicted suitable (sq-km)	Estimated Eumycetoma Cases		
			No.	95% CI	
				Lower Bound	Upper Bound
North Darfur	Um Kadada	7,578	264	33	1,002
North Kurdufan	Bara	8,245	1,192	198	4,105
North Kurdufan	Jebrat al Sheikh	17,284	654	82	2,448
North Kurdufan	Sheikan	7,590	815	113	2,963
North Kurdufan	Sowdari	7,363	521	69	1,892
North Kurdufan	Um Rawaba	12,146	1,498	234	5,174
Northern	Addabah	623	54	7	198
Northern	Dongola	1,470	524	89	1,749
Northern	Merawi	2,797	321	51	1,107
Northern	Wadi Halfa	1,806	90	12	342
Red Sea	Halayeb	2,407	23	2	92
Red Sea	Port Sudan	509	290	48	995
Red Sea	Sinkat	1,578	12	1	48
Red Sea	Tokar	3,607	467	51	1,864
River Nile	Abu Hamad	4,082	353	45	1,319
River Nile	Ad Damer	1,769	187	35	603
River Nile	Al Matammah	2,539	580	111	1,819
River Nile	Atbara	8,357	658	105	2,292
River Nile	Berber	3,380	298	47	1,022
River Nile	Shendi	7,276	655	121	2,121
Sennar	Ad Dinder	6,469	911	155	3,065
Sennar	Sennar	9,458	3,301	810	9,416
Sennar	Singa	12,446	834	145	2,790
South Darfur	Buram	743	63	8	242
South Darfur	Id El Ghanem	1,640	180	23	672
South Darfur	Kas	111	16	2	63
South Darfur	Nyala	10,013	1,081	166	3,828
South Darfur	Tulus	662	207	25	794
South Kurdufan	Abu Jubaiyah	2,351	90	14	313
South Kurdufan	Dilling	6,802	310	40	1,156
South Kurdufan	Kadugli	1,791	36	6	122
South Kurdufan	Rashad	5,420	276	36	1,026
South Kurdufan	Talodi	33	1	-	4
West Darfur	Al Geneina	5,983	506	73	1,834
West Kurdufan	Abyei	375	33	6	106
West Kurdufan	As Salam	5,212	191	26	697
West Kurdufan	En Nuhud	12,846	786	101	2,943
West Kurdufan	Ghebeish	9,743	628	79	2,361
West Kurdufan	Lagawa	6,783	212	26	803

State	District	Area predicted suitable (sq-km)	Estimated Eumycetoma Cases		
			No.	95% CI	
				Lower Bound	Upper Bound
White Nile	Ad Douiem	8,766	2,240	455	7,011
White Nile	Al Gutaina	8,035	1,711	352	5,204
White Nile	Al Jabalian	6,586	947	177	3,035
White Nile	Kosti	17,468	2,265	404	7,584
**Total**		**365,304**	**51,541**	**9,893**	**166,073**
