# Supplementary material for: Estimating the burden of mycetoma in Sudan for the period 1991–2018 using a model-based geostatistical approach
Source: PLoS Negl Trop Dis. 2022 Oct 14;16(10):e0010795. doi: 10.1371/journal.pntd.0010795 (PMC9604875; doi:10.1371/journal.pntd.0010795)
Supplement: S2 Table — (PDF) [file pntd.0010795.s012.pdf]

S2_Table. Estimation of actinomycetoma cases by district in Sudan since 1991

State	District	Area predicted suitable (sq-km)	Estimated Actinomycetoma Cases		
			No.	95% CI	
				Lower Bound	Upper Bound
Al Jazirah	Al Kamlin	1,613	142	65	270
Al Jazirah	Al Mahagil	3,554	277	104	599
Al Jazirah	East al Gazera	3,938	220	90	457
Al Jazirah	North al Gazera	2,950	357	158	692
Al Jazirah	Sharq al Gazera	5,278	329	127	715
Al Jazirah	South al Gazera	2,861	188	59	466
Al Jazirah	Um Al Gura	2,801	288	116	595
Al Qadarif	Al Faw	9,503	49	9	160
Al Qadarif	Al Fushqa	7,734	86	19	250
Al Qadarif	Al Gadaref	2,759	65	26	141
Al Qadarif	Al Galabat	2,493	26	5	83
Al Qadarif	Al Rahd	11,036	263	70	707
Blue Nile	Ad Damazin	1,673	77	22	208
Blue Nile	Al Roseires	659	41	11	110
Blue Nile	Baw	9	-	-	-
Central Darfur	Mukjar	946	31	4	115
Central Darfur	Zallingi	1,904	58	12	174
East Darfur	Al Deain	1,750	60	21	145
East Darfur	Nyala	3,911	124	24	398
Kassala	Al Gash	3,755	61	14	175
Kassala	Hamashkorieb	816	23	5	70
Kassala	Kassala	2,932	97	35	222
Kassala	Nahr Atbara	4,913	131	37	347
Kassala	Seteet	3,612	55	12	164
Khartoum	Karary	1,231	107	35	274
Khartoum	Khartoum	346	126	75	199
Khartoum	Khartoum Bahri	3,433	390	172	795
Khartoum	Omdurman	1,104	222	112	404
Khartoum	Sharg En Nile	7,705	307	89	845

State	District	Area predicted suitable (sq-km)	Estimated Actinomycetoma Cases		
			No.	95% CI	
				Lower Bound	Upper Bound
Khartoum	South Khartoum	981	123	62	219
Khartoum	Um Badda	1,110	76	35	146
North Darfur	Al Fasher	4,664	196	59	519
North Darfur	Kabkabiya	1,107	128	26	386
North Darfur	Kutum	1,901	43	12	122
North Darfur	Mellit	1,096	62	13	195
North Darfur	Um Kadada	997	20	3	69
North Kurdufan	Bara	6,435	284	94	700
North Kurdufan	Jebrat al Sheikh	15,944	364	82	1,080
North Kurdufan	Sheikan	13,377	758	185	2,149
North Kurdufan	Sowdari	9,899	559	144	1,543
North Kurdufan	Um Rawaba	24,085	861	242	2,242
Northern	Addabah	869	131	31	369
Northern	Dongola	2,030	235	67	595
Northern	Merawi	1,848	151	39	396
Northern	Wadi Halfa	187	10	2	32
Red Sea	Halayeb	10	-	-	-
Red Sea	Port Sudan	393	67	24	145
Red Sea	Sinkat	1,711	13	2	49
Red Sea	Tokar	435	43	6	158
River Nile	Abu Hamad	2,094	82	14	271
River Nile	Ad Damer	1,197	49	17	112
River Nile	Al Matammah	2,938	225	77	512
River Nile	Atbara	6,255	183	46	526
River Nile	Berber	2,985	84	25	211
River Nile	Shendi	8,096	246	81	588
Sennar	Ad Dinder	5,267	151	45	382
Sennar	Sennar	9,719	418	158	922
Sennar	Singa	10,232	160	44	418
South Darfur	Buram	429	16	3	50

State	District	Area predicted suitable (sq-km)	Estimated Actinomycetoma Cases		
			No.	95% CI	
				Lower Bound	Upper Bound
South Darfur	Id El Ghanem	1,289	44	9	134
South Darfur	Nyala	6,495	228	66	619
South Darfur	Tulus	683	69	13	211
South Kurdufan	Abu Jubaiyah	1,524	17	3	59
South Kurdufan	Dilling	2,782	44	8	138
South Kurdufan	Kadugli	220	1	-	4
South Kurdufan	Rashad	4,972	99	22	295
West Darfur	Al Geneina	4,368	143	40	393
West Kurdufan	Abyei	69	1	-	2
West Kurdufan	As Salam	4,264	54	11	162
West Kurdufan	En Nuhud	8,058	291	68	856
West Kurdufan	Ghebeish	7,149	160	31	501
West Kurdufan	Lagawa	3,372	39	7	129
White Nile	Ad Douiem	9,832	394	112	1,030
White Nile	Al Gutaina	8,035	295	108	654
White Nile	Al Jabalian	6,361	143	47	343
White Nile	Kosti	18,235	324	89	880
**Total**		**323,218**	**12,284**	**3,800**	**31,296**
